# Ultrasound imaging with an electric stimulant was useful in pulsed radiofrequency for chronic knee pain in the medial region

**DOI:** 10.1186/s40981-022-00585-6

**Published:** 2022-12-02

**Authors:** Satoshi Shimizu, Narihito Iwashita, Sei Fukui, Hirotoshi Kitagawa

**Affiliations:** 1 Department of Anesthesiology, Shiga University of Medical Science, Setatsukinowa-Cho, Otsu City, Shiga Prefecture Japan; 2 Department of Pain Management Clinic, Shiga University Hospital, Setatsukinowa-Cho, Otsu City, Shiga Prefecture Japan

To the Editor

Several studies have reported the successful palliation of knee osteoarthritis-associated chronic pain using pulsed radiofrequency (PRF) targeting of the genicular or saphenous nerve [1, 2]. Ultrasound-guided interventional treatment of these peripheral nerves usually requires identification of landmarks such as bony structures, soft tissue, and vessels, because these peripheral branches are barely identifiable [3, 4]. Moreover, the peripheral branches responsible for chronic knee pain are difficult to identify the genicular branch of the tibial nerve or the peripheral branches of the saphenous nerve [5]. In this case, we successfully identified the infrapatellar branch of the saphenous nerve as the cause of chronic knee pain in the medial knee joint region using ultrasound imaging in combination with electric stimulation.

An 83-year-old man had received weekly hyaluronic acid injections into the right knee joint for approximately 3 months at the orthopedic clinic, with unsuccessful outcomes. The patient had been consulting the Pain Management Clinic Department of our institute for approximately 12 years for postherpetic neuralgia in the left fifth and sixth thoracic vertebral regions and requested treatment for right knee pain. The patient had been prescribed 20 mg of duloxetine hydrochloride, 10 mg of myrogabalin besilate, and 1 mg of fentanyl citrate patch for postherpetic neuralgia. He complained of burning pain in the superior medial knee joint region, which exhibited mild edema, with a numeric rating scale of 7–8/10. A bilateral knee radiograph indicated mild osteoarthritis with Kellgren and Lawerence grade 1. We speculated the involvement of neuropathic pain and decided to perform PRF. Ultrasound scanning of an especially painful point revealed a cord-like structure between the vastus medialis and sartorius muscle (Fig. 1). After subcutaneous anesthesia, a guiding needle was inserted near the structure under ultrasound guidance and successfully obtained reproducible pain with less than 0.5 V of electric stimulation. The reproducible pain was obtained only when the needle tip was within a few millimeters of the cord-like structure, indicating that the structure was the peripheral branch of a nerve. PRF was performed below 42 °C for 180 s, followed by the administration of 0.45 mg of 0.15% ropivacaine and 4 mg of betamethasone. Thirty minutes after the procedure, the pain in the medial part of the right knee joint disappeared. After 1 week, he presented mild pain with a numeric rating scale of 2–3/10 at the medial knee joint region, and the edema had resolved. Three sessions of PRF successfully palliated the knee pain.

**Fig. 1:**
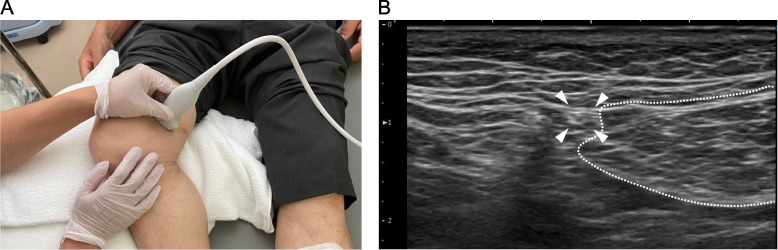
Ultrasound imaging at an especially painful point. **A** Ultrasound pre-scanning of an especially painful point in the superior medial knee joint region. **B** Ultrasound scanning reveals a cord-like structure (arrow heads) anterior to the sartorius muscle (surrounded by the white dashed line)

Several reports have described successful pain management of chronic neuropathic knee pain with PRF. These reports proposed several landmarks to identify the peripheral nerve branches [1, 2]; however, due to individual anatomical variations in innervation, the responsible nerve cannot be easily identified based only on physical findings [5]. Ultrasound imaging combined with an electrical stimulation to confirm reproducible pain could be a potential method for precisely identifying the responsive branch [6]. Such a careful diagnosis may help demonstrate the effectiveness of PRF targeting of peripheral nerves.

## Data Availability

Not applicable.

## Abbreviation

PRF: Pulsed radiofrequency
